# Suchian Feeding Success at the Interface of Ontogeny and Macroevolution

**DOI:** 10.1093/icb/icw041

**Published:** 2016-06-01

**Authors:** Paul Gignac, Haley O’Brien

**Affiliations:** *Department of Anatomy and Cell Biology, Oklahoma State University Center for Health Sciences, Tulsa, Oklahoma 74107-1898, USA; ^†^Department of Biological Sciences (Graduate Program in Ecology and Evolutionary Biology), Ohio University, Athens, Ohio 45701, USA

## Abstract

There have been a number of attempts to explain how crocodylian bite-force performance covaries with cranial form and diet. However, the mechanics and morphologies of crocodylian jaws have thus far remained incongruent with data on their performance and evolution. For example, it is largely assumed that the functional anatomy and performance of adults tightly fits the adult niche. At odds with this precept are groups with resource-dependent growth, whose juvenile stages undergo shifts in mass, morphology, and resource usage to overcome strong selection related to issues of small body size, as compared to adults. Crocodylians are an example of such a group. As living suchians, they also have a long and fossil-rich evolutionary history, characterized by analogous increases in body size, diversifications in rostrodental form, and shifts in diet. Here we use biomechanical and evolutionary modeling techniques to study the development and evolution of the suchian feeding apparatus and to formally assess the impact of potential ontogenetic-evolutionary parallels on clade dynamics. We show that patterns of ontogenetic and evolutionary bite-force changes exhibit inverted patterns of heterochrony, indicating that early ontogenetic trends are established as macroevolutionary patterns within Neosuchia, prior to the origin of Eusuchia. Although selection can act on any life-history stage, our findings suggest that selection on neonates and juveniles, in particular, can contribute to functionally important morphologies that aid individual and clade success without being strongly tied to their adult niche.

## Introduction

Suchia (Reptilia: Archosauria; *sensu* Butler et al. 2011; Nesbitt 2011) is a 240-million-year-old clade primarily comprised of predatory taxa, represented today by the crown clade Crocodylia (alligators, caimans, crocodiles, and the Indian and Malay [“false”] gharials; Gatesy et al. 2004). Crocodylians stand out among living vertebrates for their exceptional absolute bite forces, which can reach higher than 16,000 N in the largest forms (*Crocodylus porosus*; Erickson et al. 2012). There have been a number of attempts to explain how crocodylian performance covaries with cranial form and diet (Busbey 1995; McHenry et al. 2006; Pierce et al. 2008); however, the mechanics and morphologies of their jaws have remained incongruent with data on performance and evolution (Erickson et al. 2012, 2014).

Extant adult crocodylian rostrodental morphotypes are commonly considered ecomorphs (Brochu 2001), broadly divisible into four categories: taxa with rostra that are (1) slender (e.g., *Crocodylus johnsoni*, *Gavialis gangeticus*), (2) intermediate (e.g., *Alligator mississippiensis*, *C. crocodylus*), (3) blunt (e.g., *Caiman latirostris*, *Osteolaemus tetraspis*), or (4) dorsoventrally vaulted (e.g., *Paleosuchus palpebrosus*, *P. trigonatus*). As ecomorphs, the functional arrangement of the adult feeding system is thought to directly facilitate performance metrics that tightly fit the adult niche (Wainwright 1988; Norton et al. 1995). Based on this paradigm, attributes for niche-specific prey capture, such as skull strength, rostral bending, and maximum bite-force capacity, have long been expected to covary (Busbey 1995; McHenry et al. 2006; Pierce et al. 2008; see Erickson et al. 2012 for elaboration). This covariation is thought to be central to an understanding of crocodylian ecology, as rostrodental performance should physically dictate the range of food resources available to each ecomorph (*sensu* Arnold 1983). In general, this is consistent with adult prey preferences: slender-snouted forms typically feed on small, compliant prey (Pooley 1989; Webb and Manolis 1989; Thorbjarnarson 1990); forms with intermediate rostra take nearly any available invertebrate and vertebrate taxon (Pooley 1989; Webb and Manolis 1989); blunted-rostrum ecomorphs consume durable food resources like mollusks (Pooley 1989; Webb and Manolis 1989; Borteiro et al. 2009); and the paleosuchids, with the dorsoventrally deepest rostra, hunt primarily terrestrial prey (e.g., porcupines, snakes; Pooley 1989; Webb and Manolis 1989; Magnusson and Lima 1991).

Surprisingly, however, recent experimental work by Erickson et al. (2012, 2014) has challenged this ecomorphological paradigm by documenting that crocodylian bite-forces do not vary according to rostral phenotype. Instead, measured performance reflects only differences in body size, even after accounting for phylogenetic relatedness. This pattern suggests that many species—particularly those with smaller adult body sizes or slender rostra—are over-performers (*sensu* Carrier 1996), capable of generating (1) kilogram-for-kilogram bite forces that are comparable to those of morphologically robust taxa, which secure vastly different prey items (Grenard 1991; Erickson et al. 2014), and (2) adult tooth pressures far in excess of the ultimate shear strength of cortical bone (the most durable prey tissues processed by crocodylians; Erickson et al. 2012; Gignac and Erickson 2015). Although these results are non-intuitive when considering rostrodental morphology, their clade-wide ubiquity may result from two factors. First, there is strong conservation in the size and anatomical configuration of the jaw-closing musculature, which is relatively invariant across nearly all species examined to date (Iordansky 1964; Schumacher 1973; Sinclair and Alexander 1987; Busbey 1989; Cleuren et al. 1995; Endo et al. 2002; Holliday and Witmer 2007; Bona and Desojo 2011). Second, because crocodylians undergo resource-dependent growth, the majority of selection on the feeding apparatus is unlikely to be concentrated on adult phenotypes. Instead, homogeneous performance across adult ecomorphs may, in fact, be shaped by selective pressures placed on juveniles. Even in cases of male mate competition, in which jaw clapping, mock biting, and forceful biting act as a signals of social dominance (Garrick and Lang 1977), the highest bite forces of competing adults are already established through trajectories of ontogeny.

Outside of Crocodylia, it is also largely assumed that adult forms tightly fit their adult niche (L’Héritier and Teissier 1935; Carrier 1996; Loreau 2000; Pocheville 2014). However, once morphology and performance are quantified, this assumption can appear inconsistent, especially in taxa with resource-dependent growth (e.g., reptiles, fishes) that pass through significant selective filters during ontogeny (Vincent et al. 2007; Erickson et al. 2014; Herrel et al. 2016). For these taxa, the functional morphology of adults may better reflect selection for juvenile performance, rendering functional extremes in mature ecomorphs that do not couple with their preferred niche (i.e., due to ontogenetic inertia; Gignac and Santana 2016). This incongruence is well-exemplified by extant suchians, which have extreme performance capacities (for generating bite-forces) and strong selection winnowing early ontogenetic stages. Although crocodylians are long-lived species (> 25 years; Grenard 1991; Erickson and Brochu 1999), they have type III survivorship curves (Webb and Manolis 1989; Abercrombie et al. 2001). This equates to an incredibly small percentage of each cohort surviving to successful reproduction, with most small-sized individuals succumbing to environmental factors (e.g., flooding, drought, exposure), competition for food resources, or predation by larger animals (Abercrombie 1989; Abercrombie et al. 2001). Survivorship beyond the first year is limited to 20% of a hatching cohort. Only about 5% of the original generation survives to sexual maturity, and that value is halved again for those few individuals that reproduce successfully (Webb and Manolis 1989; Abercrombie et al. 2001). Survivorship is ultimately achieved by those that reach larger body sizes faster.

Against this backdrop of strong selection, neonate and juvenile crocodylians must undergo several major dietary transitions in their efforts to reach the safety of large body sizes. All species begin life as opportunistic feeders, foraging largely on insects (Grenard 1991). During their first year of life, however, juveniles rapidly gain access to a wider range of small prey items such as fish, frogs, small reptiles, and crustaceans. As the jaws and teeth approach the adult configuration (at ∼≥ 90 cm total length; Gignac and Erickson 2015), individuals become able to consume more robust prey such as birds, small mammals, and mollusks. With increasing body sizes brings the additional capability of subduing large game (e.g., deer, wild boar). Finally, the most massive individuals are able to feed on the most durable prey, capable of crushing even the thick, bony armor of turtles (Dodson 1975; Grenard 1991; Abercrombie et al. 2001; also see Fig. 5 in Erickson et al. 2003). Thus, not only does racing to a larger body size put an individual at a selective advantage, but the very race itself is won via an exponential increase in bite forces, rendering mature adult jaws that are more forceful than necessary.

However, when the evolution of suchia is considered, repeated convergence of rostrodental phenotypes throughout the history of the clade would appear to support the hypothesis that selection has acted on adult rostrodental patterns. The conspicuousness of rostral convergence has been a major focus of evolutionary biomechanists, who tend to test hypotheses about how selection has targeted adult ecomorphologies specifically (Daniel and McHenry 2001; McHenry et al. 2006; Pierce et al. 2008; Erickson et al. 2012; Walmsley et al. 2013). Nevertheless, some aspects of suchian evolution seem to mirror ontogeny. For example, there is substantial overlap in ontogenetic and evolutionary body-size trends. Living crocodylians are unique among extant tetrapods for undergoing several-thousand-fold increases in body size during ontogeny (Webb et al. 1983; Britton et al. 2012). A similar size increase is also seen spanning adults of the earliest fossil suchians, like the diminutive *Gracilisuchus* of the Early Triassic, to the gigantic salt water crocodile of today (*C. porosus*; Webb et al. 1983; Britton et al. 2012). This ontogenetic-evolutionary overlap extends to shifts in craniofacial robustness, reconstructions of the jaw-closing musculature, tooth form, and diet. Since we now know that bite force is demonstrably independent of snout shape in modern crocodylians, which pattern—adult ecomorphology or ontogenetic inertia—is supported when only the mechanical drivers of bite force are examined evolutionarily?

In this study, we seek to formally compare ontogenetic and evolutionary allometry of bite-force correlates to help resolve this apparent discrepancy between performance data and ecomorphological convergence. We used only bite-force-relevant osteological proxies that were derived from models of how bite forces are generated in living suchians. This allowed us to evaluate shifts in performance along both evolutionary and ontogenetic progressions. If adult over-performance reflects heavy selection on juvenile performance, then this should appear in the fossil record as more rapid evolutionary rates for bite force when compared to rates of body-size evolution (Klingenberg 1998; Bonduriansky and Day 2003). A pronounced establishment of such evolutionary heterochrony would signal the importance of positive bite-force allometry in suchian evolution, irrespective of repeated segregation of skull shapes into well-defined ecomorphologies. Using phylogenetic comparative methods, we tested the hypothesis that ontogenetic allometry of bite forces in living suchians mirrors patterns of evolutionary rate changes across their long diversification. We identified the onset of evolutionary heterochrony near the origin of Eusuchia, suggesting that modern patterns of ontogeny can be dated to within Neosuchia. Our findings inform how historical changes in development can manifest as shifts in performance when framed in the context of functional heterochrony, ontogenetic inertia, and evolutionary legacy.

## Materials and methods

To examine trends in suchian bite-force evolution, we combined pre-existing biomechanical analyses with phylogenetic comparative methods. We identified osteological proxies for bite-force capacity and documented the developmental and evolutionary histories of these structures. We used these to model the strength of evolutionary patterns of bite-force changes, which were then compared to bite-force allometry during ontogeny.

### Phylogenetic analysis

We generated a Bayesian, time-calibrated phylogeny of Suchia to serve as a basis for comparative analysis. We used the character matrix of Turner and Sertich (2010), which contains 81 taxa and 301 discrete morphological characters. Time calibration was performed using first-and-last occurrence data downloaded from the Fossilworks Database (see Fossilworks References in Supplementary Material). This analysis is intended to provide meaningful branch lengths calibrated by both time and character change. For consideration of interspecies relationships, see Turner and Sertich (2010), Pol et al. (2014), and Turner (2015). For further details regarding our phylogeny-building protocol, see Supplementary Material.

### Osteological proxies of bite-force performance and body size

Identification of osteological proxies for bite-force performance involved developing a functional-anatomical model of bite-force generation in *A. mississippiensis* (Gignac and Erickson 2016, forthcoming). To ensure ontogenetic applicability, this model was tested against measured maximum bite-forces for a developmental series of the same taxon (Gignac and Erickson 2016, forthcoming). Of the osteological elements involved in bite-force generation, the anterior–posterior length of the retroarticular process (RAP) was identified as correlating strongly to measured maximum bite forces in living crocodylians, regardless of ontogenetic stage (Pearson’s product moment correlation = 0.97; Supplementary Table S5). Within the crocodylian jaw adductor system, the RAP is the insertion for the two most massive muscles (*Musculus pterygoideus dorsalis* and *ventralis*; Lakjer 1926; Holliday and Witmer 2007), such that RAP length acts as the anatomical in-lever through which 60–70% of bite-force generation is transmitted (see Table 6 in Gignac and Erickson 2016, forthcoming). RAP length, therefore, was selected as the focal osteological proxy of our models. To account for body size, we measured head width (HW) across the quadrate-articular joint, as this measurement correlates strongly to body mass among extant crocodylians (Pearson’s product moment correlation = 0.99; Supplementary Table S6). To account for large differences in body size among suchians, the natural logs of all measurements were used for subsequent evolutionary and ontogenetic modeling. For complete details on measurement protocols and criteria, see Supplementary Material.

### Specimen sampling

We measured RAP and HW in a developmental series of wild *A. mississippiensis* (28–364 cm total length; *n* = 34). Each specimen was captured alive either by nuisance alligator hunters licensed through the Florida Fish and Wildlife Conservation Commission (FWC) in Leon and Jackson Counties, FL, by FWC researchers in Alachua and Marion Counties, FL, or by research staff of the Louisiana Department of Wildlife and Fisheries in Cameron Parish, LA. Animal protocols were approved by the Animal Care and Use Committees of the Florida State University, Tallahassee, FL, USA (Permit Number: 0011) and Stony Brook University, Stony Brook, NY, USA (Permit Number: 236370-1). For details on measuring RAP length in living animals, see Supplementary Material. No animals were injured during this research.

We identified fossil taxa from across our suchian phylogeny that were complete enough for evolutionary analysis (*n* = 36). These specimens have fully preserved RAP lengths and HWs. Specimens were sourced from museum collections (Supplementary Table S7) or figures available in the literature (Supplementary Table S8). The diversity of suchians with available RAP and HW data is spread evenly across the phylogeny (early Suchia *n* = 4/12; Notosuchia *n* = 13/29; Neosuchia *n* = 18 [Neosuchia A *n* = 8/15; Neosuchia B *n* = 10/22]) and represents the full range of known suchian feeding ecologies, including piscivores, generalist predators, hypercarnivores, omnivores, and herbivores. Species not represented in the morphological dataset were pruned from the original phylogeny (see Supplementary Fig. S1).

### Modeling evolutionary tradeoffs

To identify shifts in evolutionary rates, we modeled the changes in RAP length relative to body size throughout the evolution of Suchia. All calculations were performed in R, using the packages “evomap,” “phytools,” “ape,” “diversitree,” and “geiger” (respectively: Smaers 2014; Revell 2012; Paradis et al. 2004; FitzJohn 2012; Harmon et al. 2008). This first involved assessing the fit of standard models for continuous phenotypic character change and identifying the presence and probability of shifts in the adaptive landscape of RAP length and bite forces. These preliminary analyses are described in detail in the Supplementary Material. According to the best-fit model of character change (Supplementary Table S1), we derived ancestral character states and evolutionary rates for both RAP and HW, using an adaptive peak, multiple variance Brownian motion (mvBM) model (adaptive peak: Smaers and Vinicius 2009; mvBM: Smaers et al. 2016). This model allows variable rate estimation on individual branches, which renders it well suited for modeling evolution of traits that are subject to multiple selective pressures (Smaers and Vinicius 2009; Smaers et al. 2012; Smaers and Soligo 2013; Goswami et al. 2014; Smaers et al. 2016). The flexibility offered by a model that infers variable evolutionary rates helps mitigate the inaccurate tracing of character history inherent in methods that rely on a single evolutionary model (e.g., directional, Brownian motion or single-optimum Ornstein-Uhlenbeck; Schluter et al. 1997; Garland et al. 1999; Oakley and Cunningham 2000; Webster and Purvis 2002; Finarelli and Flynn 2006). The mvBM model uses a Bayesian Markov chain Monte Carlo resampling protocol (10,000,000 iterations) to estimate ancestral states at internal nodes and to quantify rates of morphological change along each branch (Smaers et al. 2012, 2016). For complete details of evolutionary model fitting and selection, see Supplementary Material.

**FIG. 1:**
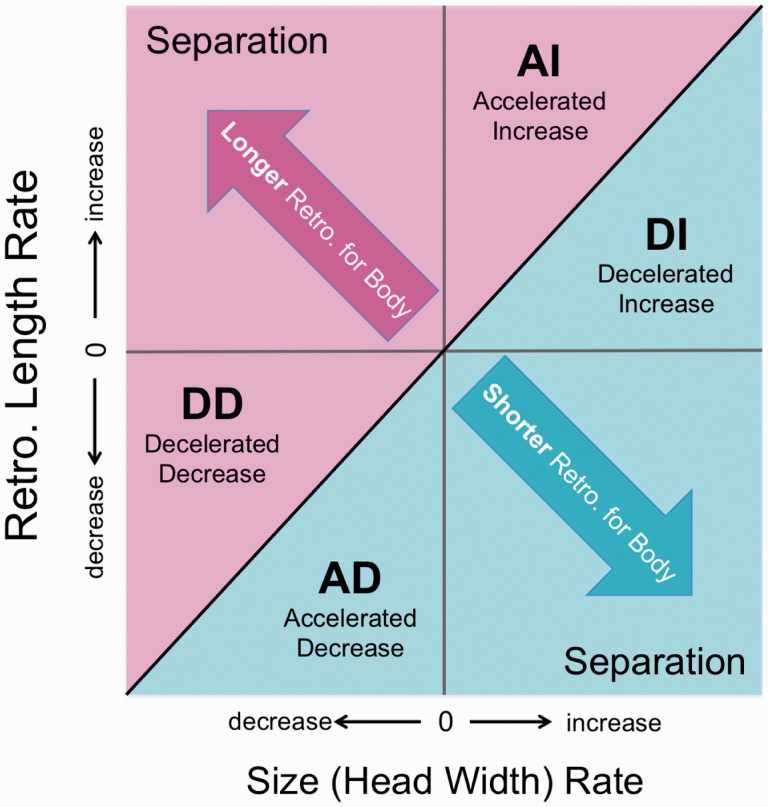
Evolutionary rate-space diagram, illustrating evolutionary tradeoffs between RAP length and body size (using HW as a proxy) change. The top left half of the plot indicates relative increase in RAP length, and, therefore, higher bite-forces. The bottom right half indicates relative decrease in RAP length, and therefore lower bite-forces. Because this study investigated the drivers of bite-force increase, negative shifts in RAP length are not considered here. There are three scenarios that indicate a positive departure from allometry: decelerated decrease (DD), whereby RAP length is decreasing more slowly than body size; separation, whereby the RAP elongates while body size decreases; and AI, whereby RAP length is increasing faster than body size. Of these, AI most closely mirrors the strongly positive changes seen in bite-force across ontogeny. Schematic redrawn from Smaers et al. (2012).

**FIG. 2:**
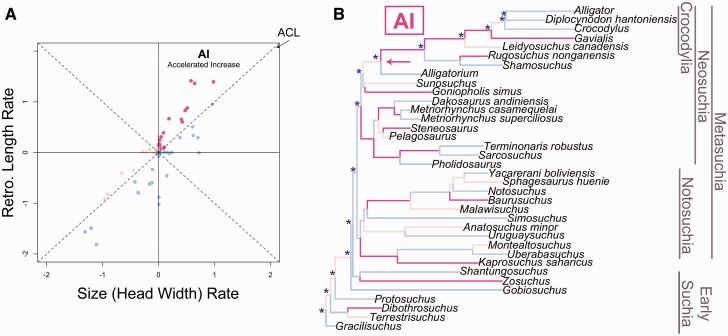
(**A**) Evolutionary tradeoffs in RAP lengths and HW for Suchia, with species falling into each evolutionary scenario. The AI (hot pink points [darkest points in grayscale]) quadrant has the highest density of data points (*n* = 17) and represents branches across which RAP-length increases are faster than body-size increases. In the context of suchian bite-force evolution, this quadrant houses instances of disproportionately positive bite-force increase—an evolutionary scenario that broadly mirrors the underlying ontogenetic allometry of bite-force increase. (**B**) Phylogeny of Suchia pruned to include fossil taxa with complete RAP length and HW data (*n* = 36). Extant representatives of crown Crocodylia include *Alligator*, *Crocodylus*, and *Gavialis*. *Gracilisuchus* is the most basal suchian in the phylogeny. Branch colors correspond to the evolutionary rate tradeoff scenarios described in the “Materials and Methods” section and figured in (A), with blue indicating a smaller RAP for a given body size and pink indicating a larger RAP for a given body size. The hot pink branches (darkest lines in grayscale) represent AI of RAP length relative to body size, indicating evolutionary stages characterized by strongly positive RAP allometry. The arrow represents the onset of a continuous AI trend from the most recent common ancestor of *Alligatorium* and *Alligator* (node no. 65 in Supplementary Fig. S2), leading to extant Crocodylia (node no. 69 in Supplementary Fig. S2). The asterisks (*) along the backbone of the tree represent the series of nodes ancestral to extant *Alligator*. RAP length and HW were estimated for all nodes, and values indicated by an asterisk (*) were divided into 10 progressive stages, which are presented in Fig. 3 and Supplementary Table S9.

Following rate and character value estimations, we conducted reduced major axis regression of RAP length and HW evolutionary rates (Smaers et al. 2012). The residuals of these rates were then plotted against each other to create a plot of relative evolutionary rate space (Fig. 1; Smaers et al. 2012). This rate space represents tradeoffs encapsulated by six possible evolutionary scenarios (Fig. 1). Disproportionately higher bite forces (when compared to body size) can be achieved under three scenarios: (1) accelerated increase (AI), whereby the RAP increases faster than body size; (2) decelerated decrease, whereby the RAP decreases slower than body size; and (3) complete separation of the traits, such that pure RAP elongation is achieved. Conversely, a relatively lower bite forces can be achieved under inverse scenarios—a pattern not identified for direct ancestors of extant suchians. These relative changes for each trait are then mapped onto each branch in the phylogeny (Fig. 2; Smaers and Vinicius 2009). For this study, only AI is considered, as this scenario represents the most rapid, positive bite-force changes (i.e., the scenario that most closely mirrors bite-force allometry throughout development).

### Ontogenetic versus evolutionary heterochrony

Heterochrony in an evolutionary context manifests as divergent trends in trait comparisons across evolutionary and ontogenetic scales (Gould 1977, 1992; Klingenberg 1998; McKinney and McNamara 1991). Ontogenetic inertia stands to unite proximate developmental patterns of heterochrony with evolutionary shifts that occur in deep time using a framework that promotes the impacts of the former on outcomes the latter. In this study, evolutionary heterochrony was modeled using the values for RAP length and HW calculated from ancestral character estimation (described above). We considered each direct ancestral node for *Alligator* (indicated by asterisks in our phylogeny, Fig. 2; labeled by node number in Supplementary Fig. S2). Including measurements for *Alligator*, 15 ancestral values were derived (Supplementary Table S9). For comparison between ontogenetic and evolutionary heterochrony, ratios for average RAP:HW (each as natural log values to account for orders-of-magnitude differences in size) were plotted across 10 sequential stages (Supplementary Table S10). These stages are arbitrary to a degree for the ontogeny dataset, due to the indeterminate growth of crocodylians. For the evolutionary dataset, each progressive stage represents stepwise transitions within the suchian phylogeny, with the earliest ancestral nodes in bin 1 and extant *Alligator* in bin 10 (Supplementary Table S9). We used a ratio of two linear measurements to facilitate more direct comparisons of heterochrony between our ontogenetic and evolutionary datasets, and because any increase in the value of RAP:HW is indicative of a relatively longer RAP, and, therefore, relatively higher bite-force capacities.

## Results

**FIG. 3:**
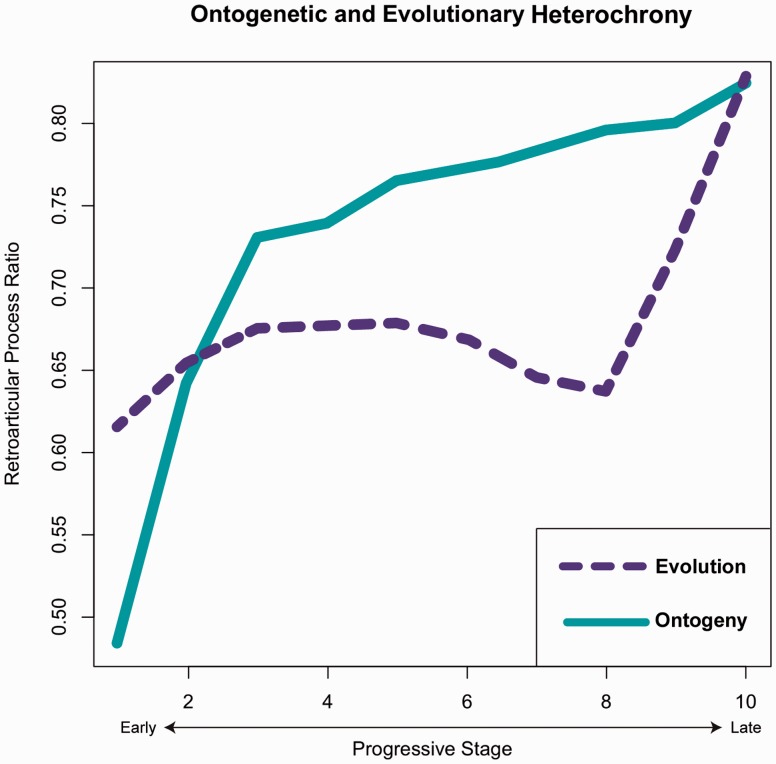
Values for RAP length as a ratio of HW plotted across 10 progressive stages. The dashed line represents the evolutionary trajectory of RAP change, derived from ancestral character estimations for RAP length and HW. The solid line represents the ontogenetic trajectory of RAP length in *Alligator mississippiensis* (a representative crocodylian; Erickson et al. 2014). A ratio of RAP:HW was utilized to facilitate comparisons between fossil adults and the developmental series of extant neonates, juveniles, and adults, which may not be directly comparable based on size alone. Both evolutionary and ontogenetic bite-force trends can be characterized as heterochronic processes.

In terms of bite-force evolutionary rates, there are taxa that fall into each tradeoff category (Fig. 2A). Lineage-specific evolutionary rates prior to and within Eusuchia, in particular, were dominated by AIs in RAP length (Fig. 2B). We found that RAP:HW ratios can be characterized by heterochronic patterns on both developmental and evolutionary timescales although the pattern is inverted (Fig. 3). For ontogenetic heterochrony, RAP allometry rapidly increases during early developmental stages, followed by decelerated RAP growth into maturity. The direct ancestors of *Alligator* demonstrate conservation of relative RAP growth along most ancestral stages, with a pronounced acceleration starting in advance of the basal node for Eusuchia (see Supplementary Material for prior probabilities; Supplementary Table S4).

## Discussion

The results of our analyses suggest that two major trends describe the evolution of suchian bite-forces along the lineages leading to extant *Alligator*: (1) stabilization of RAP length during the group’s early diversification until, (2) after the origin of Neosuchia, which demonstrates a period of AI in RAP length from prior to Eusuchia up to, and including, crown Crocodylia. When mapped together, our findings do not support the hypothesis that ontogenetic and evolutionary allometries mirror each other. Instead, we see broad support for positive allometry only within Neosuchia, indicating that RAP heterochrony appears fairly late within the evolutionary progression of taxa.

### Evolutionary rate tradeoffs

In any evolutionary study it is important to consider morphological tradeoffs to ensure that erroneous conclusions are not drawn from apparent trait changes. In this case, variation in relative RAP length could be interpreted as the result of selection acting on bite forces specifically. However, RAP length is also tightly related to body size, another highly adaptive variable, particularly when it comes to predation. It was, therefore, essential to our interpretations of bite-force change that we addressed how RAP length changes over time while also accounting for body size. To parse out the effects of body size on our interpretations of bite-force evolution, we employed a model that quantified all possible evolutionary scenarios between these two traits (Smaers et al. 2012). Within Suchia, each scenario is represented. Here, we focus on taxa with accelerated RAP-length increase (“AI” in Fig. 2) because this hemisphere represents the strongest positive RAP elongation—essentially an evolutionary scenario that mimics the ontogenetic allometry of crocodylian bite force. When considered along the phylogeny, isolated instances of accelerated RAP-length increase are seen for the branches that lead to hypercarnivorous taxa and marine piscivores. Within Neosuchia, these instances of AI are not isolated, however. There is a pronounced trend of strongly positive evolutionary allometry along the entire lineage leading to living Crocodylia (Fig. 2).

### Ontogenetic and evolutionary heterochrony

Ontogenetic and evolutionary trait changes exist on substantially different timescales. To overcome this obstacle, we plotted mean RAP:HW ratios on the *Y*-axis, against progressive stages of ontogeny and evolution along the same *X*-axis. The *X*-axis was subdivided into 10 successive stages determined by either body size (ontogeny) or phylogenetic node depth (evolution).

Ontogenetically, much of the relative change in RAP length occurs early in development, with a pronounced increase up to Stage 3 (solid line, Fig. 3). The magnitude of this shift is likely concentrated in early ontogeny because selection on juveniles for increasing bite-force is strong, and Stage 3 is approximately the point at which *A. mississippiensis* is able to access robust prey (e.g., birds, mammals) for the first time (Gignac and Erickson 2015). Subsequently, there is a deceleration in RAP growth for the remainder of ontogeny. For evolutionary changes (dashed line, Fig. 3), we see an inverted pattern, showing conservation of relative RAP length during much of the clade’s diversification. As with the ontogeny data, there is a strong inflection point, which occurs evolutionarily late at Stage 8. This stage corresponds to node no. 66 (Supplementary Fig. S2), which is the node immediately prior to Eusuchia.

Taken together, these two different patterns imply that AI in bite-force capacity relative to body size is a heterochronic process that may be an effect of early ontogenetic pressures established near the emergence of Eusuchia. This reveals that directional shifts in evolutionary and ontogenetic bite-force maximization began more than 150 million years ago (Turner 2015), suggesting that strongly positive bite-force allometry is a distinctive, if not defining, feature of the clade. Contemporaneous to the emergence of this modern performance ontogeny is the origin of the “eusuchian palate,” in which the internal choanae are enclosed within the ventral lamina of the bony pterygoids, isolating the oral and nasal cavities by means of a fully ossified secondary palate (Turner and Buckley 2008). Such functional partitioning is thought to have strengthened the rostrum (Busbey 1995) and indicates eusuchian commitment to a semi-aquatic, ambush-predator lifestyle that would have necessarily included multiple dietary transitions during ontogeny (Brochu 2003). In addition, selection in most taxa against small body sizes, such as those typical of extinct adults and extant juveniles, may have driven developmental patterns that now distinguish eusuchians from their suchian ancestors. The resultant effect of this bite-force heterochrony with simultaneous cranial reinforcement appears to be the unapologetic over-performance of modern adults.

### Ontogenetic inertia

Our results indicate that selection on early life-history stages may be the factor that has most-impacted the evolution of eusuchian bite force. As RAP length was enhanced, descendent juveniles gained the advantage of outperforming their precursors at earlier life stages. Eusuchians, however, are long-lived (e.g., Erickson and Brochu 1999). They continue to grow throughout their lives, and as much as 70% of growth can occur after sexual maturity (Webb and Manolis 1989). Thus, adults become bite-force over-performers due, in part, to ontogenetic trends that are established in early life. When paired with growth beyond sexual maturity, the subsequent body-size increases of normal development effectively carry adult performance capabilities well beyond what would be expected or needed to occupy the adult niche. Thus, evolution seems to have promoted neonate and juvenile morphologies that, in this case, aided in individual and clade success without being strongly tied to an adult niche.

As a result of this work, we recommend that researchers be aware of potential means for identifying ontogenetic inertia in their own study systems. In the case of Crocodylia, resource-dependent growth and a type III survivorship curve set up conditions for selection to play a disproportionate role in early ontogeny. Simultaneously, it may be possible to identify critical performance thresholds that put unique demands on the success of early ontogenetic stages (Herrel and Gibb 2006; Herrel et al. 2016), such as tooth-pressure thresholds for failing prey tissues (Gignac and Erickson 2015) or endurance levels during tetanic muscle contraction (Gignac 2010). If such barriers are strong enough, they may act as selective filters for neonates and juveniles. Finally, over-performers—as originally conceived by Carrier (1996)—may be more indicative of stronger selection on early ontogenetic stages than for such factors acting directly on adults.

## Supplementary Material

Supplementary Data

## Supplementary data

Supplementary Data available at *ICB* online.
