# Supplementary material for: Suchian Feeding Success at the Interface of Ontogeny and Macroevolution
Source: Integr Comp Biol. 2016 Jun 1;56(3):449–58. doi: 10.1093/icb/icw041 (PMC4990708; doi:10.1093/icb/icw041)
Supplement: Supplementary Data [file supp_56_3_449__index.html]

Suchian Feeding Success at the Interface of Ontogeny and Macroevolution — Suchian Feeding Success at the Interface of Ontogeny and Macroevolution — Supplementary Data 

# Suchian Feeding Success at the Interface of Ontogeny and Macroevolution

## Supplementary Data

files

- Supplementary Data - pdf file
