# Supplementary material for: Suchian Feeding Success at the Interface of Ontogeny and Macroevolution
Source: Integr Comp Biol. 2016 Jun 1;56(3):449–58. doi: 10.1093/icb/icw041 (PMC4990708; doi:10.1093/icb/icw041)
Supplement: Supplementary Data [file supp_icw041_icb-2016-0032-File006_TCaccepted.pdf]

Detailed Methods

Phylogenetic Analysis

To generate a phylogeny to serve as a basis for comparative analysis, we began with the character matrix of Turner and Sertich, 2010. The original character matrix includes 81 taxa and 301 discrete characters. The total dataset contains approximately 39% missing data, which is well within the threshold for returning an accurate phylogeny (Wiens, 2003).

We used a Bayesian phylogenetic analysis to infer suchian relationships. This analysis was performed using the software MrBayes v. 3.2.2 (Ronquist et al., 2011). We followed the general protocols outlined by recent paleontological studies for deriving topologies from a morphological dataset (Müller and Reisz, 2006; Clarke and Middleton, 2008; Prieto-Marquez, 2010; Kear and Barrett, 2011; Lee and Worthy, 2012; Lee et al., 2014; Gorscak et al., 2014). We first used an Mk likelihood model of morphological evolution (Lewis, 2001). We then performed an analysis using a variable rates character evolution model. All characters were treated as unordered, and minimum age constraints (relaxed clock) were applied to each taxon using data from the Fossilworks Database (see Fossilworks References for first-appearance datum/last-appearance datum [FAD/LAD] below), which we also cross-checked against the original references. Under the variable rates model character rate variation was drawn from a gamma distribution. The Markov chain Monte Carlo setting ran for 20,000,000 generations, with sampling occurring every 1,000 generations using two heated chains. Our 25% burn-in was sufficient as

convergence was reached early in the run. The majority-rule consensus tree was generated from the combined post-burn-in samples (Supplemental Fig. S1).

This analysis is intended to provide meaningful branch lengths calibrated by both time and character change. We recovered > 90% of the topological relationships in the source tree (Turner and Sertich, 2010), with large polytomies resolved. For careful consideration of interspecies relationships, we refer the reader to Turner and Sertich (2010), Pol et al. (2014), and Turner (2015).

Morphological Data Collection

Due to variable preservation quality, four techniques were used to measure head width (HW). (1) as trans-quadratic width, for specimens with complete post-orbital skulls; (2) twice the distance between the midsagittal plane (as defined by an anteroposterior line passing through the midpoint of the occipital condyle) and the lateral margin of the quadrate, for distorted or incomplete cranial specimens; (3) trans-articular width for specimens lacking complete skulls but possessing lower jaws; and (4) or as twice the distance between the midsagittal plane (as defined by the anteroposterior midline passing through a fully articulated mandibular symphysis) and the lateral margins of the articular, for distorted or incomplete lower-jaw specimens.

Retroarticular processes (RAPs) were measured as the anteroposterior length of the articular bone from the midpoint of the quadrate articular joint (i.e., center of rotation) to the posterior-most margin of the articular bone, perpendicular to an imaginary line passing through the center of rotation of the jaw joints. Values for *Metriorhynchus casamequelai* were substituted with values for *Metriorhynchus brachyrhynchus*

(Supplemental Table S3) in the analysis of trait history as they are congeners. RAP length was measured directly on fossil skulls and was measured following manual palpation on living specimens, which were restrained to ensure measurement accuracy.

Preliminary Evolutionary Model Fitting

Prior to the evolutionary analysis described in the main text, we first fit a series of standard models of trait evolution and evaluated the location and probability of evolutionary regime shifts on the pruned phylogeny. Evolutionary models of continuous trait evolution were fitted using the 'geiger,' 'phytools,' and 'evomap' R packages (Harmon *et al.*, 2008; Revell, 2012; Smaers, 2016). Lambda (Pagel, 1999), delta (time dependent model; Pagel, 1999), kappa (punctuational model; Pagel, 1999), early burst (Harmon *et al.*, 2010; similar to the accelerating-decelerating model of Blomberg *et al.*, 2003), Brownian motion (Felsenstein, 1973), Ornstein-Uhlenbeck (random walk with central tendency; Butler and King, 2004; with variance-covariance matrix transformation of Slater, 2014), and independent evolution (adaptive peak of Smaers and Vinicius, 2009; implemented in evomap) were fit to the pruned phylogeny and values of natural-logged retroarticular process lengths (as ln mm). The phylogeny was independently scaled to each model and the posterior distribution of traits was calculated using a Bayesian Markov Chain Monte Carlo (MCMC) ancestral character estimation (2,000,000 generations). The relative goodness-of-fit of each model was compared using log likelihood and the small-sample-corrected Akaike information criterion (AICc). The best fit model is one of independent evolution (Supplemental Table S1), which was, therefore, employed in the analysis described in the main text. The worst fit model was Ornstein-

Uhlenbeck (Supplemental Table S1). This may be due to the fact that our pruned phylogeny is both non-ultrametric and contains a sample size fewer than 200 taxa. These issues are described in detail by Slater (2014) and Cooper and colleagues (2016).

SUPPLEMENTAL TABLE S1. Support for each model fit based on log-likelihood and AICc values. The best fit model is one of independent evolution (the adaptive peak model of Smaers and Vinicius, 2009). Abbreviations: BM, Brownian Motion; EB, Early Burst; IE, Independent Evolution; OU, Ornstein Uhlenbeck.

**Model**	**Log Likelihood**	**AICc**
BM	-68.5	139.1
lambda	-58.7	121.8
delta	-67.1	138.5
kappa	-72.9	150.2
EB	-67.6	139.6
OU	-123.8	251.9
IE	-29.2	62.8

Evolutionary Model Support

Although the independent evolution model is the best fit and was used to calculate evolutionary rates and ancestral character estimations, a series of exploratory analyses was conducted to (1) estimate and (2) test hypotheses regarding the strength, directionality and probability of regime change within the tree. To identify shifts without *a priori* hypotheses, the SURFACE model of Ingram and Mahler (2013) was implemented in the R package ‘surface’ (Ingram and Mahler, 2013). SURFACE uses stepwise algorithms to locate regime shifts on a tree with the performance of each model

tested using sequential, iterative changes in AICc values (a model initially conceived of by Hansen, 1997; Ingram and Mahler, 2013). This model involves forward and backward passes, with the forward pass establishing the number of regime shifts and the backward pass identifying instances of convergence in trait evolution (Ingram and Mahler, 2013). Model output includes estimations of the parameters K (number of regime shifts), α (evolutionary rate), σ^2 (magnitude of uncorrelated diffusion), and n optima of θ (optimum trait values). Our SURFACE models accounted for both HW and RAP (in ln mm), and identified four regimes in RAP evolutionary rates: (1) early Suchia + Notosuchia (excluding hypercarnivorous Notosuchia), (2) hypercarnivorous Notosuchia, (3) Neosuchia, and (4) the clade of stem Neosuchians comprised of *Rugosuchus* + *Shamosuchus*. SURFACE estimated parameters are presented in Supplemental Table S2.

SUPPLEMENTAL TABLE S2. Regime shift parameters for suchian head width (HW) and retroarticular process (RAP) evolution (in ln mm), calculated using the Hansen method as implemented in the R package 'SURFACE' (Ingram and Mahler, 2013). Abbreviations: α, evolutionary rate; σ^2, magnitude of uncorrelated diffusion; HW, head width; K, number of regime shifts; θ_n, optimum trait values; RAP, retroarticular process length.

**Parameter**	**HW**	**RAP**
α	0.045	0.038
σ^2	0.017	0.028
K	6	4
θ_1	3.995	2.033
θ_2	5.683	4.649
θ_3	4.895	3.515

Support for the SURFACE hypothesized regime shifts was quantified using a Bayesian reversible-jump MCMC, multiple-peak Ornstein-Uhlenbeck model,

implemented using the ‘bayou’ package in R (Uyeda and Harmon, 2014). Whereas SURFACE treats α and σ^2 as constant throughout the tree, the multi-peak OU model allows these values to change. We ran two models for retroarticular process evolution in ‘bayou’. In the first, all prior parameters were unconstrained (‘heuristic priors’ below). In the second, we set our priors to be roughly equivalent to the results of our SURFACE models (‘four-shift’ below). We identified four branches on which *a priori* regime shifts were expected, introduced three values for θ (2.033, 3.515, and 4.649 ln mm), and established initial evolutionary rates and the magnitude of uncorrelated diffusion (α = 0.038, σ^2 = 0.028). Both of our ‘bayou’ models ran for 2,000,000 generations, allowed for a maximum of one regime shift per branch, and all prior distributions were assumed to be normal (with the exception of the distribution of shifts, for which the prior distribution was assumed as conditional Poisson).

Our four-shift model marginally out-performed the ‘heuristic’/unconstrained priors model (lnl = -46.76 and -49.6, respectively; summary statistics in Supplemental Table S3), with both returning high probabilities of regime shift on branches leading to (1) Metasuchia, (2) small-bodied notosuchians, (3) Neosuchia, and (4) Eusuchia (Fig. S3). The four-shift ‘bayou’ model returned a bimodal set of trait optima (Supplemental Table S4): small RAP length (below 2.5 ln mm) and large RAP length (approximately 4 to 5.5 ln mm). Trait optima, along with regime-shift posterior probabilities, are visualized in Supplemental Fig. S3. The density distribution of RAP length values is further visualized in Supplemental Fig. S4, alongside color-coded phylogenetic regime shifts with a posterior probability higher than 0.2. A regime shift toward small-size optima is moderately well-supported for Notosuchia (to the exclusion of the carnivorous taxa

Montealtosuchus, Uberabasuchus, and *Kaprosuchus*, which exhibits a shift toward increasing size). The backbone of the tree leading directly to extant Crocodylia exhibits high posterior probability shifts toward increased RAP length at three nodes: the node demarcating Metasuchia, the base of Neosuchia, and the branch leading to stem and extant Eusuchia. Within the neosuchian ancestors of modern taxa, there is a series of low prior probability (but regular) shifts toward extant Crocodylia (Supplemental Fig. S3, Supplemental Table S4). Further, there is also a secondary regime shift toward increasing trait values elsewhere within Neosuchia, at the branch leading toward largely pelagic and marine forms (e.g. *Pelagosaurus* and *Metriorhynchus*) + hypercarnivorous forms (*Sarcosuchus* and *Terminonaris*). Ultimately, the SURFACE and 'bayou' models show strong support for gradual optimization of bite force within Neosuchia, with large shifts toward longer length optima at the bases of first Neosuchia and then either Eusuchia or Crocodylia (e.g., node no. 68 of Supplemental Figure S2). It is possible to implicate either of these locations as the onset of ontogenetic inertia (and, thus, adult over-performance); however, modern data can be most directly extended to shifts within Crocodylia.

SUPPLEMENTAL TABLE S3. Summary statistics for parameters from both 'bayou' models. Abbreviations: α, evolutionary rate; σ^2, magnitude of uncorrelated diffusion; *K,* mean number of regime shifts; $N\,\theta$, mean number of optimum trait values; θ_o, predicted RAP length (ln mm) at the base of the tree; θ_{all}, mean RAP length (ln mm) for all optima.

	**Four-Shift**	**Heuristic Priors**
Log likelihood	-46.76	-49.6
Prior	-32.12	-29.36
α	0.01	0.01
σ^2	0.03	0.04
K	5.23	4.8
$N\,\theta$	6.23	5.8
θ_o	2.17	2.36
θ_{all}	3.39	3.41

SUPPLEMENTAL TABLE S4. Posterior probabilities (PP) and magnitude of regime shifts within suchian retroarticular process length evolution based on a four-shift model.

**Branch**	**PP**	**Clade**	**Magnitude of θ**	**SE θ^2**
60	0.4245	Metasuchia	4.166	0.0124
56	0.3413	Neosuchia (base)	4.711	0.0144
33	0.2549	Neosuchia (marine)	5.387	0.0165
54	0.2195	Notosuchia (small-bodied)	2.496	0.0114
53	0.1755	Notosuchia (hypercarnivores)	3.922	0.0197
12	0.1425	Crocodylia (base)	4.52	0.023
55	0.1366	Notosuchia (all)	3.138	0.0155
34	0.111	Eusuchia	4.043	0.0214
51	0.1089	*Kaprosuchus*	4.065	0.0379
59	0.1039	*Shantungosuchus* + *Zosuchus*	1.399	0.0263

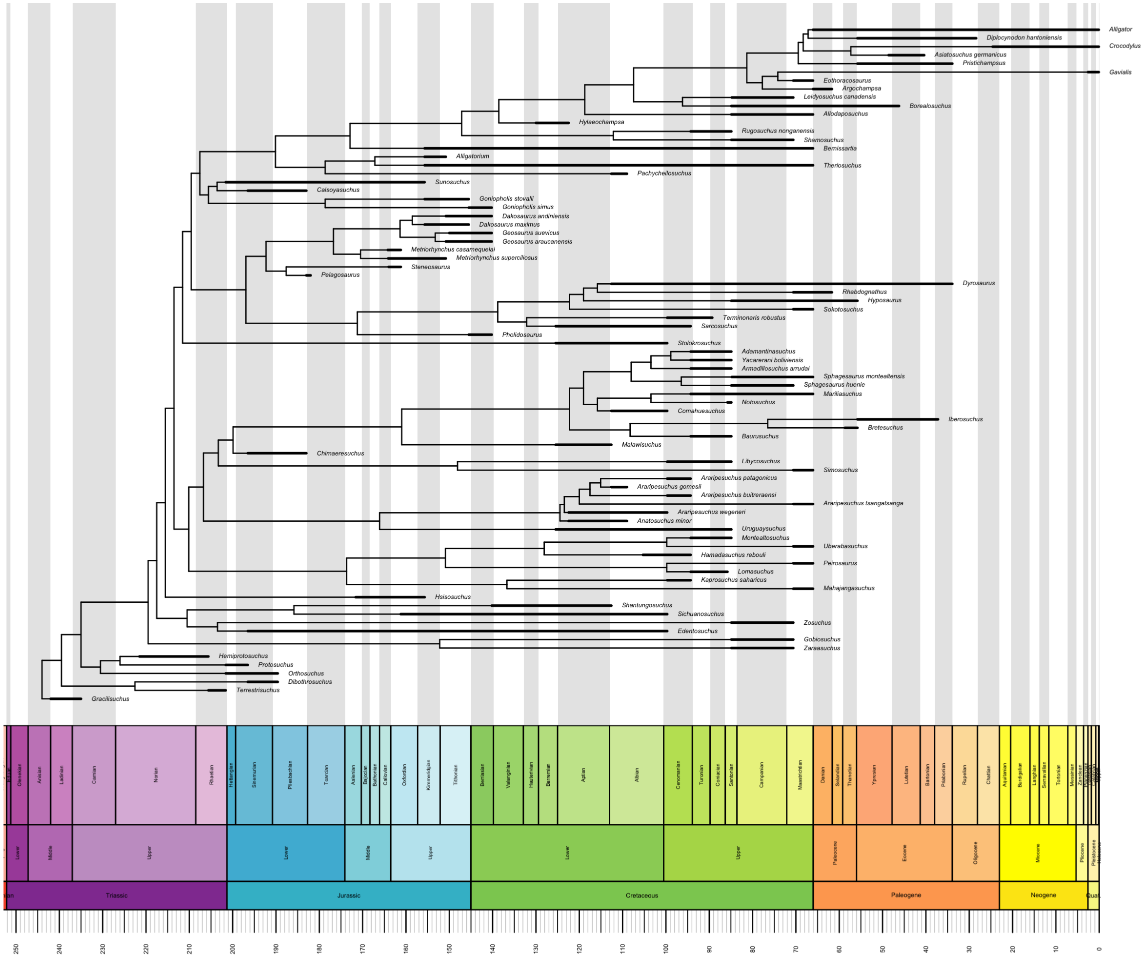

SUPPLEMENTAL FIGURE S1. Complete time-calibrated, majority-rule consensus tree of Suchia, generated from the combined post-burn-in samples. The phylogeny was derived from the character matrix of Turner and Sertich (2010). (Higher resolution version available at: https://dx.doi.org/10.6084/m9.figshare.3413596)

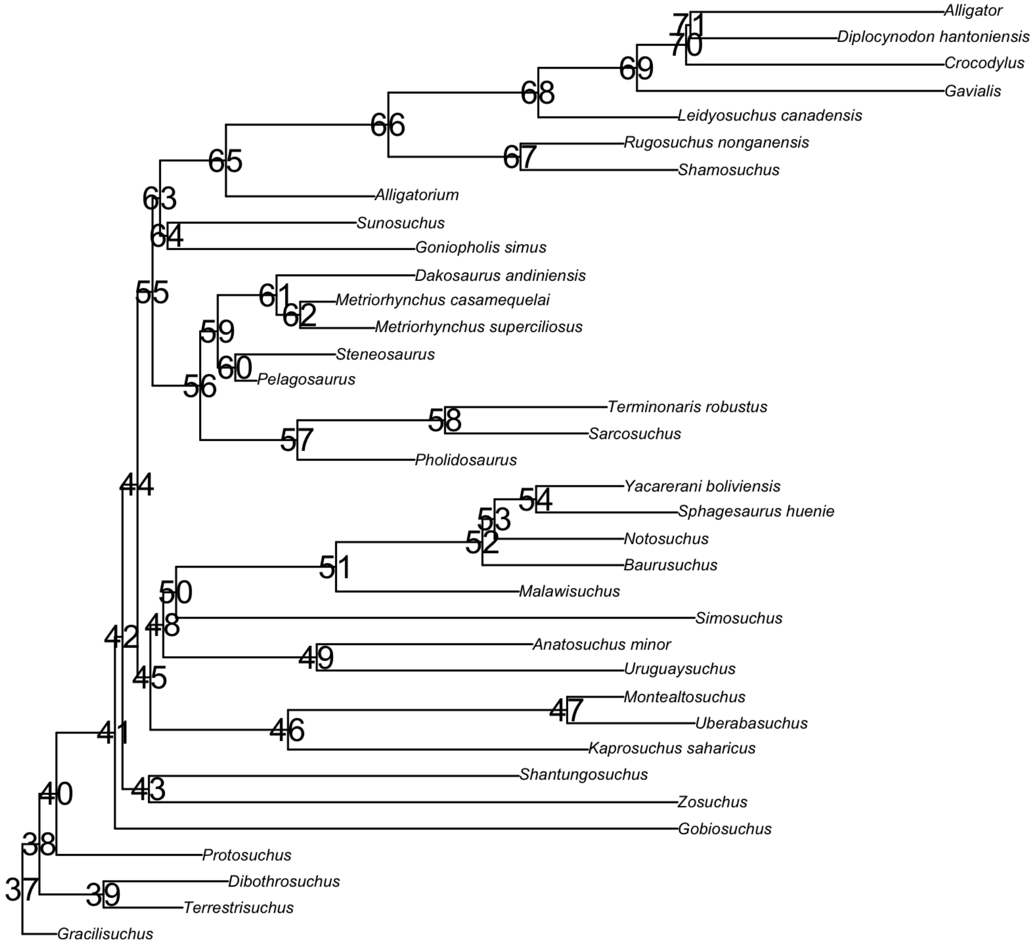

SUPPLEMENTAL FIGURE S2. Phylogeny of Suchia pruned for fossil taxa with complete retroarticular process (RAP) and head width (HW) data (n = 36) and with nodes labelled numerically (see Supplemental Table S9 for node values).

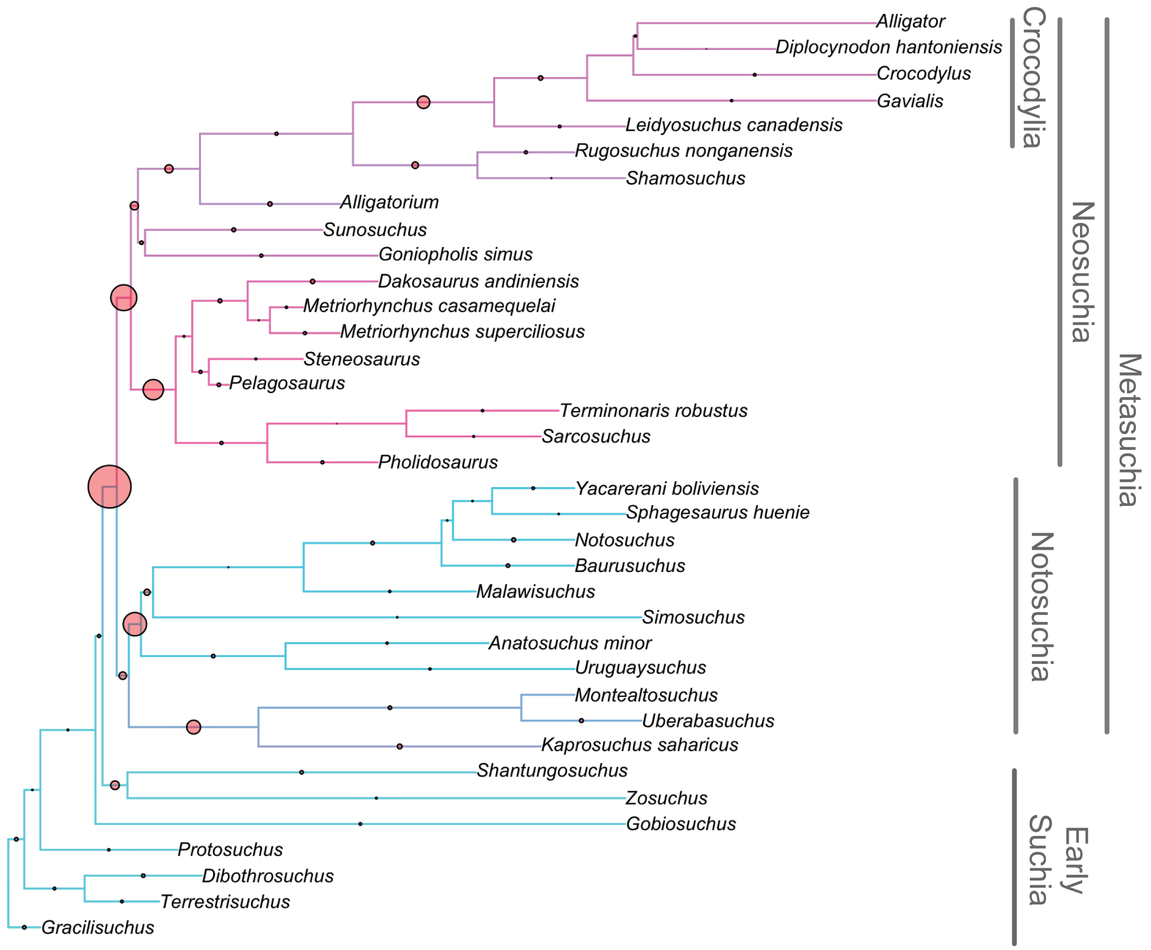

SUPPLEMENTAL FIGURE S3. Phylogeny of Suchia with retroarticular process length (bite force proxy) evolutionary regime shifts. Blue indicates shifts toward a decreasing trait value, and pink indicates shifts toward an increasing trait value. Circle sizes on branches indicate relative posterior probabilities of regime shift (larger = higher probability).

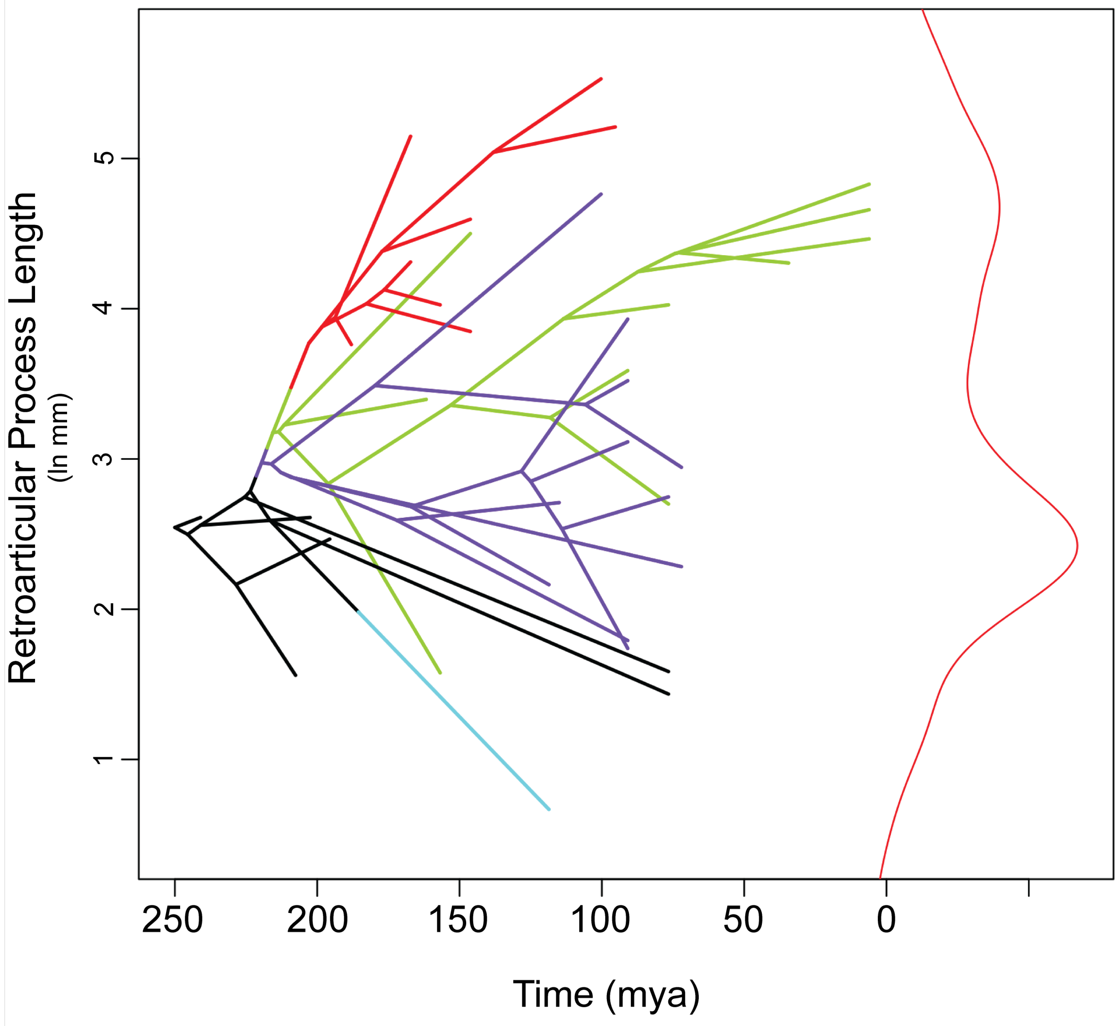

SUPPLEMENTAL FIGURE S4. Phylogeny of Suchia with tips matched to density of phenotypic trait values. Each color within the phylogeny represents a regime shift with a posterior probability of greater than 0.2. Phenotypic trait values are approximately bimodally distributed with a peak for small trait values (shorter retroarticular process [RAP] lengths and, thus, low bite forces) and a peak for higher trait values (longer RAP lengths and, thus, relatively higher bite forces). Black = early Suchia; purple = Notosuchia; green = Neosuchia (inclusive of modern Crocodylia); red = Neosuchia (e.g. marine and pelagic forms).

SUPPLEMENTAL TABLE S5. Retroarticular process (RAP) length (mm) and bite forces (Newtons; N); experimentally measured at the most prominent molariform tooth position; protocol reported in Gignac and Erickson, 2015) for a developmental series of wild *Alligator mississippiensis* (Pearson's product moment correlation = 0.97 on best-fit power regression, n = 28).

RAP	Bite Forces
4	15
5	14
9	31
10	41
20	260
24	170
25	198
27	275
28	347
35	1,076
46	1,052
50	2,323
53	2,274
55	1,780
60	2,326
62	3,512
65	3,521
66	1,884
80	4,630
80	5,900
85	8,120
90	5,840
90	5,998
95	9,618
100	10,058
110	8,833
125	11,127
126	10,057

SUPPLEMENTAL TABLE S6. Head width (HW; mm) and body-mass (kg) measurements for a developmental series of wild *Alligator mississippiensis* (Pearson's product moment correlation = 0.99 on best-fit power regression, n = 35).

**HW**	**Body Mass**
23	0.06
23	0.06
33	0.22
34	0.25
68	2.20
70	2.45
70	2.60
75	3.60
78	3.15
95	4.50
123	17.20
125	16.30
130	15.50
130	20.00
140	18.00
160	32.10
170	42.60
170	41.00
175	47.60
207	128.00
215	56.00
220	87.50
225	77.10
225	104.50
240	116.00
250	106.20
270	132.00
275	129.50
280	158.00
280	190.50
290	155.00
300	225.00
305	198.00
330	230.20

340	173.00

SUPPLEMENTAL TABLE S7. Retroarticular process (RAP) lengths (mm), head widths (HWs; mm), and specimen details for fossil taxa used in this study that were sourced from museum collections. Institutional abbreviations: **AMNH**—American Museum of Natural History, New York, USA; **FSU-VC**—Florida State University Vertebrate Collection, Florida, USA; **IGM**—Mongolian Institute of Geology, Ulaan Bataar, Mongolia; **MBR**—Museum für Naturkunde der Humboldt Universität, Berlin, Germany; **MCZ**—Museum of Comparative Zoology, Massachusetts, USA; **MVZ**—Museum of Vertebrate Zoology, California, USA; **NHM UK**—Natural History Museum, London, United Kingdom; **UA**, Université d'Antananarivo, Antananarivo, Madagascar; **UCRC**—University of Chicago Research Collection, Illinois, USA; **UFRJ**—Universidade Federal do Rio de Janeiro, Brazil.

Taxon	Specimen No.	HW	RAP
Alligator mississippiensis	FSUVC 082405-1	340	125
Crocodylus niloticus	MVZ 81487	343	106
Diplocynodon hantaniensis	AMNH 27632	224	74
Gavialis gangeticus	NHMUK 61.4.1.2	202	87
Goniopholis simus	NHMUK 5259–61	276	90
Gracilisuchus stipanicicorum	MCZ 4118	101	14
Kaprosuchus saharicus	UCRC PVC8	227	117
Malawisuchus amarali	UFRJ DG 106-R e	67	10
Metriorhynchus brachyrhynchus	UFRJ DG 105-R	273	75
Metriorhynchus superciliosis	UFRJ DG 5-R	190	56
Montealtosuchus arrudacamposi	NHMUK PV R 3804	121	34
Notosuchus	MBR 3981	135	23
Pelagosaurus sp.	MBR 2883	101	43
Pholidosaurus schaumburgensis	MBR 1965	272	99
Shamosuchus djadochtaensis	IGM 100/1105	83	15
Simosuchus clarki	UA 8670	83	10
Steneosaurus obtusidens	NHMUK PV R 3168	358	172
Zosuchus	IGM 100/1304	35	4

SUPPLEMENTAL TABLE S8. Retroarticular process (RAP) lengths (mm), head widths (HWs; mm), and specimen details for fossil taxa used in this study that were sourced from figures available in the literature. Institutional abbreviations: **BMNH**—British Museum of Natural History; **CMNH**—Carnegie Museum of Natural History; **FC-DVP**—Facultad de Ciencias, Colección de Vertebrados Fósiles, Montevideo, Uruguay; **CPPLIP**—Centro de Pesquisas Paleontológicas Llewellyn Ivor Price; **IGV**—Geological Institute of the Chinese Academy of Geological Sciences Vertebrate Paleontology Collection; **IVPP**—Institute of Vertebrate Paleontology and Paleoanthropology, Academia Sinica, Beijing; **MGB**—Museo de Geología del Ayuntamiento de Barcelona; **MNK-PAL**—Museo 'Noel Kempff Mercado'; **MNN**—Muséum National du Niger, Niamey, République de Niger; **MOZ**—Museo Profesor J. Olsacher, Zapala, Argentina; **MPMA**—Museu de Paleontologia de Monte Alto, Brazil; **MZSP-PV**—Museu de Zoologia, Universidade de São Paulo; **SMNH**—Royal Saskatchewan Museum (formerly the Saskatchewan Museum of Natural History), Regina, Saskatchewan, Canada; **ZPAL**—Instytut Paleobiologii **PAN**, Warszawa, Poland.

Taxon	Specimen No.	HW	RAP	Reference	Figure(s)
Alligatorium (*Montsecosuchus*) *depereti*	MGB 512	32	5	Buscalioni, AD, Sanz, JL. 1990. J Vert Paleo 10:244–254.	3, 4
Anatosuchus minor	MNN GAD17	60	15	Sereno PC, Larsson HCE. 2009. ZooKeys 28:1–143.	5
Baurusuchus salgadoensis	MPMA-62-0001-02	196	51	Vasconcellos FM, Carvalho IS. 2007. Paleontologia 1:319–332.	1

Dakosaurus andiniensis	MOZ 6146P	384	47	Gasparini Z, Pol D, Spalletti LA. 2005. Science 311:70–73.	1
Dibothrosuchus elaphros	IVPP V 7907	93	12	Wu X-C, Chatterjee S. 1993. J Vert Paleo 13:58–89.	4, 8
Gobiosuchus kielanae	ZPAL MgR-II 69	31	5	Osmólska H, Hua S, Buffetaut E. 1997. Act Paleo Pol 42:257–289.	6
Leidyosuchus canadensis	ROM 1903	258	63	Wu X-C, et al. 2001. Can J Earth Sci 38:1665–1687.	1, 2
Protosuchus haughtoni	BP/1/4770	46	5	Gow CE. 2000. J Vert Paleo 20:49–56.	2, 3
Rugosuchus nonganensis	IGV 33	133	36	Wu X-C, et al. 2001. Can J Earth Sci 38:1653–1663.	3, 4, 6, 7
Sarcosuchus imperator	MNN 604	840	252	Sereno P, et al. 2001. Science 294:1516–1519.	2
Shantungosuchus hangjinensis	IVPP V 10097	31	2	Wu X-C, et al. 1994. J Vert Paleo 14:210–229.	2–4
Sunosuchus miaoi	CMNH 8028	154	30	Buffetaut E, Ingavat R. 1980. Geobios 13, 879–889.	1–5
Sphagesaurus huenei (*Caipirasuchus stenognathus*)	MZSP-PV 139	70	16	Pol D, et al. 2014. PLOS ONE *9*:e93105.	22
Terminonaris robusta	SMNH P2411.1	409	183	Wu X-C, Russell AP, Cumbaa SL. 2001. J Vert Paleo. 21:492–514.	2
Terrestrisuchus gracilis	BMNH R 7557	37	5	Crush PJ. 1984. Palaeontology 27:131-157.	5
Uberabasuchus terrificus	CPPLIP 630	141	19	Carvalho I, et al. 2004. Gond Res 7:975–1002.	3

Uruguaysuchus	FC-DVP 2320	62	6	Soto M, Pol D, Perea D. 2011. Zool J Linnaean Soc 163:S173–S198.	9
Yacarerani boliviensis	MNK-PAL 5063/5064	74	6	Novas FE, et al. 2009. J Vert Paleo 29:1316–1320.	2

SUPPLEMENTAL TABLE S9. Trait histories for ratios between retroarticular process (RAP) lengths (ln mm) and head widths (HWs) (ln mm), reconstructed for each direct ancestral node between *Alligator* and the origin of Suchia (node no. 37; Supplemental Figure S1).

Node Number	HW	RAP	Stage Assignment
37	4.589067	2.812592	1
38	4.582657	2.840437	1
40	4.529138	2.876542	2
41	4.424912	2.990188	2
42	4.427167	2.991335	3
44	4.465903	3.025687	4
55	4.833527	3.286161	5
63	4.911033	3.287499	6
65	4.739929	3.058593	7
66	4.64784	2.968581	8
68	5.204641	3.306364	9
69	5.294412	4.312054	9
70	5.191259	4.314440	10
71	5.198303	4.314237	10
Alligator	5.828946	4.828314	10

SUPPLEMENTAL TABLE S10. Values for ratios between retroarticular process (RAP) lengths (ln mm) and head widths (HWs) (ln mm) for *Alligator mississippiensis*, averaged within each of ten developmental stages between hatchlings (28 cm total length [TL]) and the largest adult (364 cm TL) in our sample (n = 34).

HW	RAP	Stage Assignment
3.135494	1.386294	1
3.135494	1.609438	1
3.496508	2.197225	2
3.526361	2.397895	2
4.248495	2.995732	3
4.248495	3.178054	3
4.553877	3.218876	3
4.219508	3.218876	3
4.356709	3.295837	4
4.317488	3.332205	4
4.867534	3.367296	4
4.867534	3.555348	5
4.828314	3.688879	5
4.941642	3.806662	5
4.812184	3.828641	5
5.135798	3.912023	6
5.164786	3.951244	6
5.075174	4.007333	6
5.135798	4.094345	7
5.370638	4.127134	7
5.416100	4.174387	7
5.416100	4.248495	7
5.393628	4.290459	7
5.332719	4.382027	8
5.480639	4.382027	8
5.616771	4.382027	8
5.669881	4.442651	8
5.598422	4.499810	9
5.634790	4.499810	9
5.703782	4.553877	9
5.799093	4.653960	10
5.768321	4.700480	10
5.828946	4.828314	10
5.720312	4.836282	10
5.828946	4.828314	10

References

Blomberg S, Garland T, Ives AR. 2003. Testing for phylogenetic signal in comparative data: Behavioral traits are more labile. Evolution 57:717–745.

Buffetaut E, Ingavat R. 1980. A new crocodilian from the Jurassic of Thailand, *Sunosuchus thailandicus* n. sp. (Mesosuchia, Goniopholididae), and the palaeogeographical history of South-East Asia in the Mesozoic. Geobios 13:879–889.

Buscalioni AD, Sanz JL. 1990. *Montsecosuchus depereti* (Crocodylomorpha, Atoposauridae), new denomination for *Alligatorium depereti* Vidal, 1915 (Early Cretaceous, Spain): Redescription and phylogenetic relationships. J Vert Paleo 10:244–254.

Butler MA, King AA. 2004. Phylogenetic comparative analysis: A modeling approach for adaptive evolution. Am Nat 164:683–695.

Carvalho I, Ribeiro LCB, dos Santos A. 2004. *Uberabasuchus terrificus* sp. nov., a new Crocodylomorpha from the Bauru Basin (Upper Cretaceous), Brazil. Gond Res 7:975–1002.

Clarke JA, Middleton KM. 2008. Mosaicism, modules, and the evolution of birds: Results from a Bayesian approach to the study of morphological evolution using discrete character data. Syst Biol 57:185–201.

Cooper N, Thomas GH, Venditti C, Meade A, Freckleton RP. 2016. A cautionary note on the use of Ornstein Uhlenbeck models in macroevolutionary studies. Biol J Linn Soc 118:64–77.

Crush PJ. 1984. A late Upper Triassic sphenosuchid crocodilian from Wales. Palaeontology 27:131–157.

Felsenstein J. 1973. Maximum-likelihood estimation of evolutionary trees from continuous characters. Am J Hum Genet 25:471–492.

Gasparini Z, Pol D, Spalletti LA. 2005. An unusual marine crocodyliform from the Jurassic-Cretaceous boundary of Patagonia. Science 311:70–73.

Gignac PM, Erickson GM. 2015. Ontogenetic changes in dental form and tooth pressures facilitate developmental niche shifts in American alligators. J Zool 295:132–142.

Gorscak E, O'Connor PM, Stevens NJ, Roberts EM. 2014. A basal titanosaurian *Rukwatitan* (Dinosauria, Sauropoda) from the middle Cretaceous Galula Formation, Rukwa Rift Basin, southwestern Tanzania. J Vert Paleo 34:1133–1154.

Gow CE. 2000. The skull of *Protosuchus haughtoni*, an early Jurassic crocodyliform from southern Africa. J Vert Paleo 20:49–56.

Hansen TF. 1997. Stabilizing selection and the comparative analysis of adaptation. Evolution 51:1341–1351.

Harmon LJ, Weir JT, Brock CD, Glor RE, Challenger W. 2008. Geiger: investigating evolutionary radiations. Bioinformatics 24:129–131.

Harmon L, Losos J, Davies TJ, Gillespie R, Gittleman J, Jennings BW, Kozak K, McPeek M, Moreno RF, Near T. 2010. Early bursts of body size and shape evolution are rare in comparative data. Evolution 64:2385–2396.

Ingram T, Mahler DL. 2013. SURFACE: detecting convergent evolution from comparative data by fitting Ornstein-Uhlenbeck models with stepwise Akaike Information Criterion. Methods Ecol Evol 4:416–425.

Kear BP, Barrett PM. 2011. Reassessment of the Lower Cretaceous (Barremian) pliosauroid *Leptocleidus superstes* (Andrews, 1922) and other plesiosaur remains from the non-marine Wealden succession of southern England. Zool J Linn Soc Lond 161:663–691.

Lee MSY, Worthy TH. 2012. Likelihood reinstates *Archaeopteryx* as a primitive bird. Biol Lett 8:299–303.

Lee MSY, Cau A, Naish D, Dyke GJ. 2014. Morphological clocks in paleontology, and a Mid-Cretaceous origin of crown Aves. Syst Biol 63:442–449.

Lewis, PO. 2001. A likelihood approach to estimating phylogeny from discrete morphological character data. Syst Biol 50:913–925.

Müller J, Reisz RR. 2006. The phylogeny of early Eureptiles: Comparing parsimony and Bayesian approaches in the investigation of a basal fossil clade. Syst Biol 55:503–511.

Novas FE, Pais DF, Pol D, Carvalho I, Scanferla A, Mones A, Riglos MS. 2009. Bizarre notosuchian crocodyliform with associated eggs from the Upper Cretaceous of Bolivia. J Vert Paleo 29:1316-1320.

Osmólska H, Hua S, Buffetaut E. 1997. *Gobiosuchus kielanae* (Protosuchia) from the Late Cretaceous of Mongolia: anatomy and relationships. Act Paleo Pol 42:257–289.

Pagel, M. 1999. Inferring the historical patterns of biological evolution. Nature 401:877–884.

Pol D, Nascimento PM, Carvalho AB, Riccomini C, Pires-Domingues RA, Zaher H. 2014. A new notosuchian from the Late Cretaceous of Brazil and the phylogeny of advanced notosuchians. PLOS One 9(4):e93105.

Prieto-Marquez A. 2010. Global phylogeny of the Hadrosauridae (Dinosauria: Ornithopoda) using parsimony and Bayesian methods. Zool J Linn Soc Lond 159:435–502.

Revell LJ. 2012. Phytools: an R package for phylogenetic comparative biology (and other things). Methods in Ecol Evol 3:217–223.

Ronquist F, Huelsenbeck J, Teslenko M. 2011. MrBayes 3.2: efficient Bayesian phylogenetic inference and model choice across a large model space. Syst Biol 61:539–542.

Sereno PC, Larsson HCE. 2009. Cretaceous crocodyliforms from the Sahara. ZooKeys 28:1–143.

Sereno P, Larsson HCE, Sidor CA, Bado B. 2001. The giant crocodyliform *Sarcosuchus* from the Cretaceous of Africa. Science 294:1516–1519.

Slater GJ. 2014. Correction to ‘Phylogenetic evidence for a shift in the mode of mammalian body size evolution at the Cretaceous-Palaeogene boundary’, and a note on fitting macroevolutionary models to comparative paleontological data sets. Methods Ecol Evol 5:714–718.

Smaers JB. 2014. evomap: R package for the evolutionary mapping of continuous traits. GitHub https://github.com/JeroenSmaers/evomap.

Smaers JB, Vinicius, L. 2009. Inferring macro-evolutionary patterns using an adaptive peak model of evolution. Evol Ecol Res 11:991-1015.

Soto M, Pol D, Perea D. 2011. A new specimen of *Uruguaysuchus aznarezi* (Crocodyliformes: Notosuchia) from the middle Cretaceous of Uruguay and its phylogenetic relationships. Zool J Linn Soc Lond 163:S173–S198.

Turner AH. 2015. A review of *Shamosuchus* and *Paralligator* (Crocodyliformes, Neosuchia) from the Cretaceous of Asia. PLoS ONE 10:e0118116.

Turner AH, Sertich JJW. 2010. Phylogenetic history of *Simosuchus clarki* (Crocodyliformes: Notosuchia) from the Late Cretaceous of Madagascar. J Vert Paleo 30: S1, 177–236.

Uyeda JC, Harmon LJ. 2014. A novel Bayesian method for inferring and interpreting the dynamics of adaptive landscapes from phylogenetic comparative data. Syst Biol 63:902–918.

Vasconcellos FM, Carvalho IS. 2007. Cranial features of *Baurusuchus salgodoensis* Carvalho, Campos & Nobre 2005, A Bauruschidae (Mesoeucrocodylia) from the Adamantina Formation, Bauru Basin, Brazil: paleoichnological, taxonomic and systematic implications. Paleontologia 1:319–332.

Wiens JJ. 2003. Incomplete taxa, incomplete characters, and phylogenetic accuracy: Is there a missing data problem? J Vert Paleo 23:297-310.

Wu X-C, Brinkman DB, Lu J-C. 1994. A new species of *Shantungosuchus* from the Lower Cretaceous of Inner Mongolia (China), with comments on *S. chuhsienensis* Young, 1961 and the phylogenetic position of the genus. J Vert Paleo 14:210–229.

Wu X-C, Chatterjee S. 1993. *Dibothrosuchus elaphros*, a Crocodylomorph from the Lower Jurassic of China and the phylogeny of the Sphenosuchina. J Vert Paleo 13:58–89.

Wu X-C, Cheng Z-W, Russell AP. 2001. Cranial anatomy of a new crocodyliform (Archosauria: Crocodylomorpha) from the Lower Cretaceous of Song-Liao Plain, northeastern China. Can J Earth Sc. 38:1653–1663.

Wu X-C, Russell AP, Cumbaa SL. 2001. *Terminonaris* (Archosauria: Crocodyliformes): new material from Saskatchewan, Canada, and comments on its phylogenetic relationships. J Vert Paleo 21:492–514.

Fossilworks References for FAD/LAD (with collections and occurrences counts):

Brinkman DB. 1990. Paleontology of the Judith River Formation (Campanian) of Dinosaur National Park, Alberta, Canada: evidence from vertebrate microfossil locality. Palaeogeogr Palaeocl 78:37–54. [14 collections, 20 occurrences]

Clyde WC. 1997. Stratigraphy and mammalian paleontology of the McCullough Peaks, northern Bighorn Basin, Wyoming: Implications for biochronology, basin development, and community reorganization across the Paleocene-Eocene boundary. PhD dissertation, University of Michigan. [57 collections, 79 occurrences]

Delfino M, de Vos J. 2010. A revision of the Dubois crocodylians, *Gavialis bengawanicus* and *Crocodylus ossifragus*, from the Pleistocene *Homo erectus* beds of Java. J Vert Paleo 30:427–441. [12 collections, 20 occurrences]

Eberth DA, Brinkman DB. 1997. Paleoecology of an estuarine, incised-valley fill in the Dinosaur Park Formation (Judith River Group, Upper Cretaceous) of southern Alberta, Canada. Palaios 12:43–58. [12 collections, 13 occurrences]

Estes R. 1964. Fossil vertebrates from the Late Cretaceous Lance Formation, eastern Wyoming. Univ Cal Publ Geol Sci 49:1–180. [4 collections, 31 occurrences]

Harris JM. 1983. Koobi Fora Research Project: The Fossil Ungulates: Proboscidea, Perissodactyla, Suidae. Oxford (UK): Clarendon Press. p. 321. [10 collections, 31 occurrences]

Huene FV. 1942. Lieferungen 3/4. Pseudosuchia, Saurischia, Rhynchosauridae und Schlussabschnitt [Parts 3/4. Pseudosuchia, Saurischia, Rhynchosauridae, and

Conclusions]. In: Die Fossilen Reptilien des Südamerikanischen Gondwanalandes. Ergebnisse der Sauriergrabungen in Südbrasilien 1928/29 [The Fossil Reptiles of South American Gondwanaland. Results of the Dinosaur Expeditions in southern Brazil 1928/29]. München (GE): C. H. Beck'sche Verlagsbuchhandlung. p.161–332 [12 collections, 15 occurrences]

Krause DW, Sertich JJW, Rogers RR, Kast SC, Rasoamiaramanana AH, Buckley GA. 2010. Overview of the discovery, distribution, and geological context of *Simosuchus clarki* (Crocodyliformes: Notosuchia) from the Late Cretaceous of Madagascar. Soc Vert Paleo Mem 10:4–12. [9 collections, 18 occurrences]

Langston Jr W. 1965. Fossil crocodilians from Colombia and the Cenozoic history of the Crocodilia in South America. Univ Cal Publ Geol Sci 52:1–169. [7 collections, 17 occurrences]

Lillegraven JA, Eberle JJ. 1999. Vertebrate faunal changes through Lancian and Puercan time in southern Wyoming. J Paleo 4:691–710. [16 collections, 27 occurrences]

Long RA, Murry PA. 1995. Late Triassic (Carnian and Norian) tetrapods from the southwestern United States. New Mex Mus Nat His Sci Bull 4:1–254. [77 collections, 155 occurrences]

Lucas SG, Heckert AB, Hunt AP. 2002. A new species of the aetosaur *Typothorax* (Archosauria: Stagonolepididae) from the Upper Triassic of east-central New Mexico. In: Heckert AB, Lucas SG, editors. Upper Triassic Stratigraphy and Paleontology. New Mex Mus Nat His Sci Bull 21:221–233 [9 collections, 17 occurrences]

Pearson DA, Schaefer T, Johnson KR, Nichols DJ, Hunter JP. 2002. Vertebrate biostratigraphy of the Hell Creek Formation in southwestern North Dakota and northwestern South Dakota. Geol S Am S 361:145–167. [30 collections, 68 occurrences]

Scheyer TM, Aguilera AO, Delfino M, Fortier DC, Carlini AA, Sánchez R, Carrillo-Briceño JD, Quiroz L, Sánchez-Villagra MR. 2013. Crocodylian diversity peak and extinction in the late Cenozoic of the northern Neotropics. Nat Comm 4:1–9. [8 collections, 33 occurrences]

Westphal F. 1962. Die Krokodilier des Deutschen und Englischen Oberen Lias. Palaeontogra Abt A 118:23–118. [15 collections, 28 occurrences]

Young MT, Hua S, Steel L, Foffa D, Brusatte SL, Thuring S, Mateus O, Ruiz-Omenaca JI, Havlik P, Lepage Y, Andrade MB. 2014. Revision of the Late Jurassic teleosaurid genus *Machimosaurus* (Crocodylomorpha, Thalattosuchia). R Soc Open Sci 1:140222. [13 collections, 19 occurrences]
